# Implant therapy in patients with chronic periodontitis: A short follow-up with a successful outcome

**DOI:** 10.15171/japid.2019.007

**Published:** 2019-08-31

**Authors:** Farhan Durrani, Akanksha Shukla, Himani Painuly

**Affiliations:** ^1^Department of Periodontics and Oral Surgery, Faculty of Dental Sciences, Institute of Medical Sciences, Varanasi, Hndia

**Keywords:** Biological complications, bone loss, dental implants, long-term survival, peri-implantitis, periodontitis, supportive periodontal therapy

## Abstract

Chronic periodontitis is a progressive disease with loss of several teeth. Implant therapy in these patients can be a successful outcome as long as the tissues are kept healthy from a microbiologic viewpoint. Regular follow-up visits after complex reconstruction is the key for long-term success. In our report, recall visits were kept on short intervals for ten years. The results showed that implants were very good prosthetic replacements in chronic sufferers of the disease but regular followups are the gold standard.

## Introduction

Dental implants are regularly placed in patients suffering from chronic periodontitis. Implant treatment in periodontitis-susceptible individuals is frequently debated. It has been reported that in partially or completely edentulous patients, periodontal pathogens might be transmitted from teeth to implants, implying that periodontal pockets might serve as a reservoir for bacterial colonization. The microflora similarity of periodontitis and peri-implantitis support the concept that periodontal pathogens might be associated with peri-implantitis and failing implant. The hard and soft tissues of these patients are host-modulated and susceptible to aggravation of disease. There are certain factors which are associated with the susceptibility of these conditions. Poor oral hygiene and cigarette smoking are the strongest risk indicators. Design of the prosthesis and excess cement may also be associated. The importance of treating existing periodontitis prior to the placement of dental implants has often been emphasized. It is often said that implant prosthesis in a patient with chronic periodontitis has more chances of biological complications and failure around implants. In our study, we followed two patients with chronic periodontitis; one was fully edentulous and the other was partially edentulous. The aim of the current study was to investigate the survival of implants replacing missing teeth in subjects with chronic periodontitis for more than ten years. The primary outcome was implant survival; MBL (marginal bone loss) and PPD (probing pocket depth) were secondary parameters.

### A Partially Edentulous Patient

**Figure 1:**
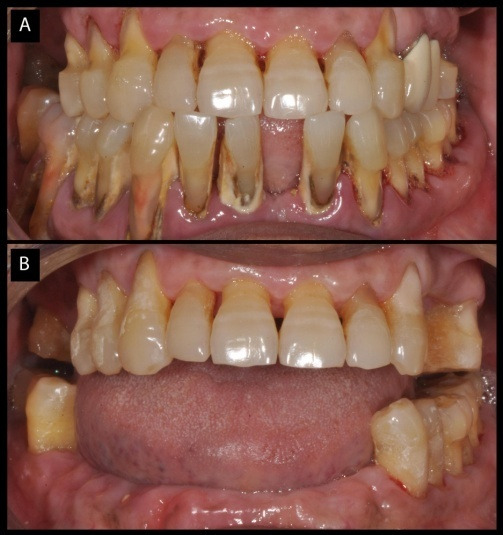
(a) Chronic periodontitis in the partially edentulous patient with doubtful prognosis of lower anterior teeth; (b) Extraction of hopeless teeth.

A 59-year-old male was referred to the Periodontic Division of Faculty of Dental Sciences for evaluation of bleeding gums and mobile teeth. There was no medical history associated, with overall good health of the patient. On dental examination, bleeding on probing (full mouth) was present with calculus on all the lower anterior teeth. Attachment loss was more than 50% of lower incisors, extending to the posterior area on the right side up to the first molar. Maxillary dentition had generalized abrasion on the anterior and posterior teeth with attachment loss of <50% and without any mobility. Teeth #16 and #17 were missing with #24 and #25 restored with PFM (porcelain fused-to-metal) crowns. The reason for poor oral hygiene was inadequate awareness and careless approach towards dental care. Radiographic examination revealed the chronic state of periodontitis. PPD ranged was 3‒9 mm with horizontal and vertical bone defects in the lower dentition. Grade 3 mobility was associated with teeth #46, #45, #44, #43, #42, #41, #31, #32 and #33 along with #24 and #25 in the maxilla. The following teeth were extracted because of doubtful prognosis (Figure 1, a and b).After healing of the sockets, the patient underwent supragingival and subgingival debridement of all the teeth. Cervical abrasions in both the upper and lower remaining teeth were restored with glass-ionomer cement (GC Fuji IX, GC Corporation, Japan). Plaque and gingival bleeding indices were evaluated periodically for the next three months. The patient exhibited significant improvements with scores <30%. An interim partial denture was provided for the patient and further advised to use 0.12% chlorhexidine rinse twice daily.

### Surgical phase

As the periodontal parameter scores improved and it was considered acceptable for oral hygiene maintenance, the patient requested fixed replacement for teeth; dental implants in strategic positions was a viable treatment plan.

### Implant planning

Diagnostic imaging and clinical evaluation were carried out for implant placement. The casts were mounted on a semi-adjustable articulator; an ideal mandibular diagnostic wax-up was achieved with minor corrections. This position was duplicated into radiographic and surgical guide for prosthetic space analysis. A screw-retained prosthesis was planned over implants with optimal position of access channels for implant screws. Angled prosthetic abutments were not ruled out in the final prosthesis. Six implants were planned in the edentulous region in the mandible and four implants for maxillary edentulous spaces.

### Implant surgery

**Figure 2:**
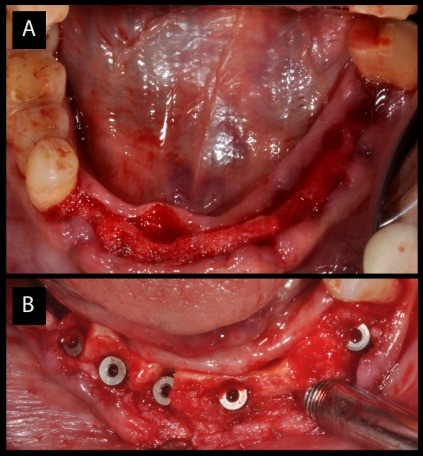
(a) Mucoperiosteal flap reflected in the partially edentulous patient; (b) Six implants, osteotomy completed.

The procedure started after complete healing of the tissue approximately four months after extraction. The patient was given adequate anaesthesia for mandibular implant surgery with bilateral inferior alveolar nerve blocks. The tissue was exposed with single incision, extending from the tooth region #34 up to #47. The surgical guide was stabilized and the resin from the lingual side of the guide was removed for complete view of the soft tissues. Mucoperiosteal flap elevation was carried out with periosteal elevators and the lingual flaps were sutured to the opposite flaps, respectively. Six implants (Rapid, Dentin Implants Technologies, Israel) were placed in the edentulous region carefully with lingual placement in extraction sockets and the guide was evaluated appropriately for best positioning. All the implants (3.3×10 – 3 nos. and 3.8×10 – 3 nos.) had thread thicknesses changing from the apex to the neck with the same pitch, improving the compression of bone during insertion. The design provided initial implant stability in the extraction sockets (Figure 2, a  and b). The buccal part of each socket was augmented with a xenograft (Bio-Oss, Geistlich Pharma, Switzerland) and covered with a collagen membrane (Biogide, Geistlich Pharma, Switzerland). The flaps were sutured in place with 4-0 ethicon sutures in a horizontal and interrupted design. There was simultaneous implant placement in the maxilla with 2 implants, with a size 3.3×10 on the left side and 3.8×8 on the right side. Cover screws were placed and primary closure was obtained over the implants and all the implants underwent submerged healing for 5 months.

### Maxillary and mandibular prosthesis

**Figure 3:**
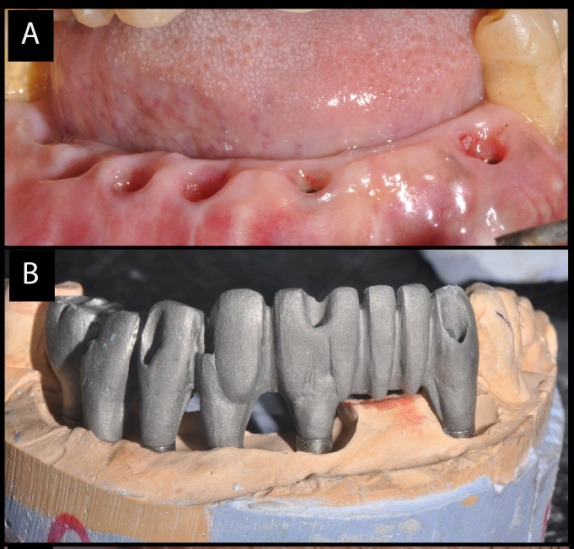
(a) Complete healing after osteotomy of six implants; (b) Framework for the prosthesis.

**Figure 4:**
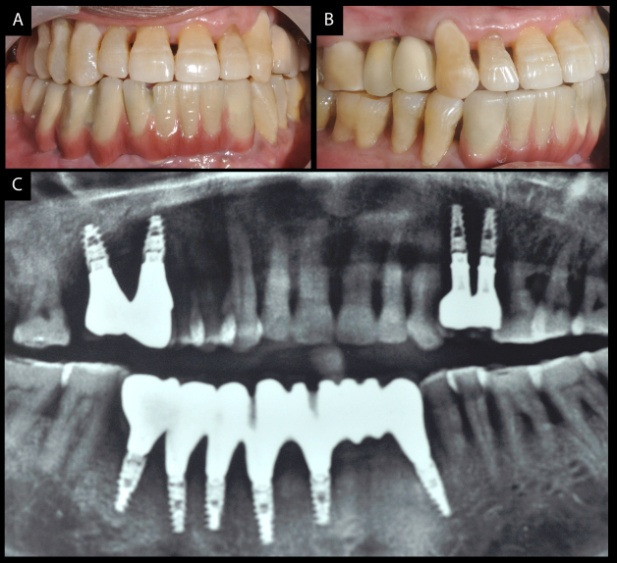
(a) Porcelain screw-retained prosthesis; frontal view; (b) Lateral view; (c) Orthopantomogram of the prosthesis.

After 5 months, all the implants were uncovered. They were evaluated for mobility, bone loss or any infection. Impression transfers were attached to all the implants and open tray impression was taken with addition silicone material (Dentsply). Each implant connection was verified with x-ray and evaluated before making the impression. All the impression transfer posts were splinted together with resin (Pattern Resin) and cut initially and then again joined to counter the expansion of the material. Definitive cast was poured in type IV stone. Diagnostic casts were prepared for both the maxilla and mandible. Standard prosthodontic principles were followed, which included maxilla-mandibular relations. As all the implants were relatively parallel, multiunit abutments were used for optimal positioning for screw access channels in the final prosthesis. A cast metal framework was fabricated over the abutments from silver palladium alloy (Figure 3, a  and b). The passivity and fit of the framework was tried in the patient mouth in both arches. Sheffield screw testing was carried out for the framework check in the mandible. Bite registration was taken and recorded (O-bite). A final prosthesis try-in was performed to confirm accurate transfer of teeth, phonetics and mutually protected occlusion. The final prosthesis was fabricated in the porcelain fused-to-metal for both arches. The occlusal screws of maxillary and mandibular prosthesis were tightened to 15 Ncm. The screw channels were filled with warm gutta-percha resin and sealed with composite resin (Figure 4, a, b  and c). The patient was given postoperative cleaning instructions using Proxa brushes and powered water irrigation system. The patient was kept on 3-month follow-up recalls for periodontal maintenance for 5 years. During these visits periodontal assessment was carried out with routine supragingival and subgingival debridement. Oral hygiene was further reinforced if needed. Prosthetic complications were assessed, too. The patient was quite satisfied with the prostheses of both arches. At 5-year recall, all the implants were stable and the prostheses were devoid of any complications. The periodontal condition of the remaining natural teeth was quite stable. The radiographs taken around implants revealed bone levels within normal limits. After 5 years, 6-month recall visits were scheduled for the next five years.

### A completely edentulous patient

**Figure 5:**
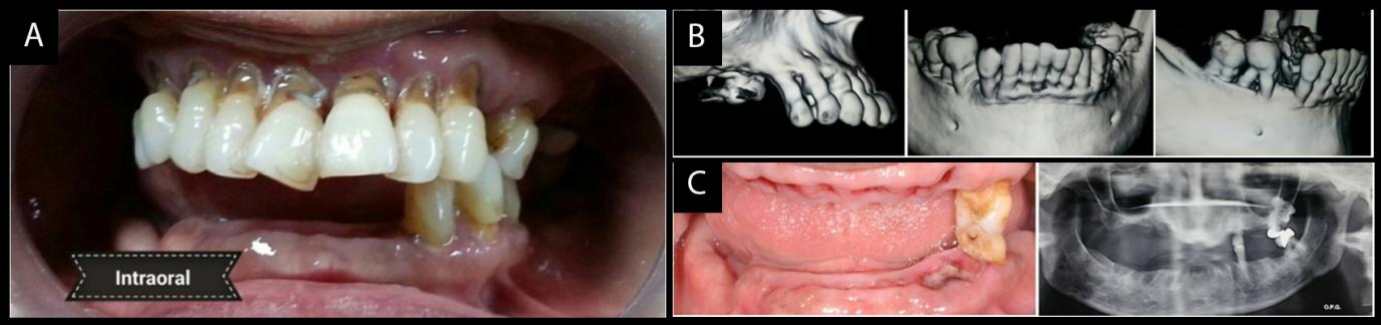
(a) Chronic periodontitis patient; (b) 3D view showing the extent of bone loss; (c) Edentulism after extraction except for premolars to maintain the vertical height.

A 65-year-old female patient presented, complaining of inability to chew properly. Although the patient was in good health without any medication, oral neglect was unaccountable. On oral clinical examination, the entire dentition seemed mobile. There was >50% attachment loss on all the teeth, with grade 3 furcation involvement in lower molars bilaterally. Complete extraction was advised, except for teeth #25 and #35 to preserve the vertical dimension of occlusion (Figure 5, a, b and c). Complete dentures were fabricated after a tissue healing period of 6 weeks. The patient was kept on 3-month follow-ups to monitor oral hygiene maintenance.

**Figure 6:**
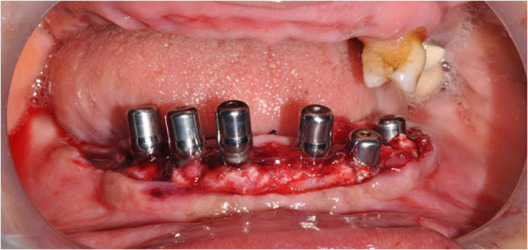
Six implants in the mandible.

**Figure 7:**
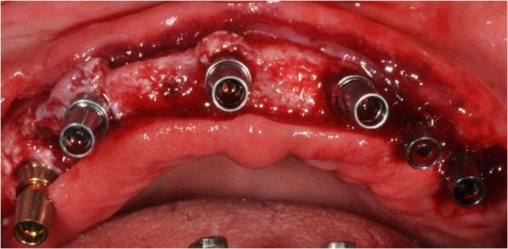
Six implants in the maxilla.

The patient agreed for fixed replacement of teeth and gave the consent for complex reconstruction for implant screw-retained prostheses for both the upper and lower arches. After four months of denture wearing and complete soft tissue healing, the dentures were duplicated in a surgical guide and the patient underwent a CBCT scan to decide optimal positions for implants in each arch. Six tissue-level implants (Myriad Connect, Equinoxmed Belgium) were placed in the mandible between the inter-foramina region (3.3/9.5 mm). The maxilla also had six implants between the maxillary sinus region (3.4/11; 2, 3.4/13; 2, 3.4/9.5;2, Xive Dentsply**) (**Figures 6 and 7). The retained second premolars of each arch were extracted during the surgery and replaced by implants. The patient had a waiting period of five months for prosthesis fabrication after surgery. The existing complete dentures were adjusted and relined several times to provide a perfect occlusion and oral hygiene was further evaluated every month.

**Figure 8:**
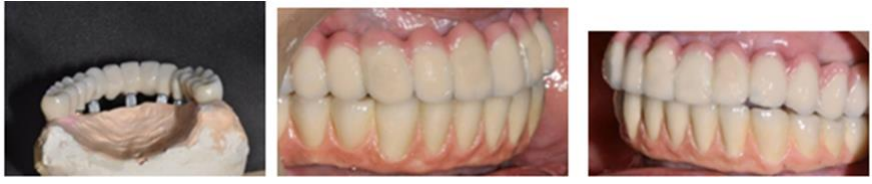
(a) PFM prosthesis; (b) Frontal view; (c) Lateral view.

Evaluation and acceptance of oral hygiene maintenance led to the fabrication of fixed prostheses. The integration of each implant was evaluated carefully with percussion and RVG x-ray. The impression of each arch was taken following standard prosthodontic principles, and milled framework was fabricated through CAD‒CAM. They were further checked on the implants for their passive fit. The metal‒ceramic screw-retained prostheses were fitted both in the maxilla and mandible. Anterior posterior spread of the prosthesis was kept in such a way that it did not cross more than 1.5 times. The occlusion was mutually protected with upper canines guiding the lateral movements. After giving the final torque to each implant, the access holes were closed with a Teflon tape and composite resin (Figure 8, a, b and c). Superfloss was advised for cleaning below the prostheses and fluoride toothpaste for cleaning the entire arch. A protective splint for upper arch was given to be worn in the night for six months. The patient was recalled every three months for evaluation of occlusion and oral hygiene for the next five years.

### Follow-up

Both subjects were clinically and radiographically monitored initially at baseline. Full-mouth plaque score, full-mouth bleeding score, PPD (probing pocket depth) and MBL (marginal bone loss) were measured at four sites (teeth) with two sites (implants) in partially edentulous and completely edentulous patients by a periodontal probe (UNC 15, Hu-Friedy, Chicago, IL, USA) and rounded off to the nearest millimetre.

At baseline, both patients were periodontally treated with a pocket depth of <3 mm, a plaque score of <30% and a bleeding score of <25%.

The following parameters were evaluated in each follow-up visit:

1. Percentage of surfaces with plaque
2. Percentage of bleeding on probing
3. Residual pockets
4. Marginal bone loss

The patients were placed on individual tailored maintenance care program (3-month recalls) for 5 years, followed by 6-months recalls for the next 5 years. Continuous progression of the disease was evaluated carefully. Reinstruction, re-instrumentation and treatment of re-infected sites were performed as needed. Cumulative interceptive supportive periodontal therapy was standardized, which included mechanical cleaning and antiseptic therapy with chlorhexidine gluconate.

After 10 years, examinations were carried out by two periodontists who were new to the cases. Full-mouth modified plaque score was calculated by a probe around marginal gingiva of the teeth and implants.

Score 0: No plaque

Score 1: Visible plaque

Score 2; Plaque visible to the naked eye

Score 3: Abundant plaque

Modified sulcus bleeding index were as follows:

Score 0: No bleeding

Score 1: Isolated bleeding

Score 2: Blood along the margin

Score 3: Profuse bleeding

Pocket depths were determined at four sites for teeth (mesial, distal, buccal and lingual) and two sites for implants and lost teeth in both patients. The distance between the implant shoulder and the most coronal visible bone-to-implant contact (DIB) was measured in millimetre at both the mesial and distal aspects of each implant using standardized (long-cone technique) periapical intraoral radiographs. Mobility was tested manually and evaluated with the Periotest instrument (Siemens AG, Germany). Based on the findings, the teeth and the implant were assessed to be successful or unsuccessful.

The scores of both the patients for plaque and bleeding were between 0 and 2 on average. Following surgery and delivery of prostheses, both patients reported no complications except for the complaint about long waiting for prosthesis fabrication. Initially, the patients had adjustment problems in occlusion, floss and proxa brushes for cleaning under the prostheses. There was peri-implant mucositis when the screw-retained prosthesis was removed for cleaning in six months. However, it was resolved after further reinforcement of oral hygiene maintenance. There was no mobility of teeth or implant in either of the prostheses in the initial follow-up of three months. The patients were adjusted with their mutually protected occlusion and seemed quite happy with the change from initial clinical presentation. The initial 3-month follow-up for five years made the patients perform optimum oral care. Mean plaque and bleeding scores remarkably improved at each follow-up. This further led to a stable oral environment for 6-month follow-up, too. Pocket depth remained around 4.2‒4.7 mm on average in both patients around the implants for the first 5 years. Radiographic findings did not reveal any continuous peri-implant radiolucencies around the implants or teeth in observation period of five years, too. The distance between the implant shoulder and the most coronal visible bone-to-implant contact (DIB) was 2.4 mm to 2.9 mm on average in five years for both patients. However, attachment loss increased in the partially edentulous patient by 1.3‒1.7 mm on the remaining teeth in spite of strict 3-month recall for five years.

After five years, the recall was scheduled for six months. All the implants in both patients remained stable, with 3.6‒4.2-mm distance between the implant shoulder and the most coronal visible bone-to-implant contact (DIB) on average after completion of ten years. However, plaque scores and bleeding index were higher as compared to the initial 5-year visits.

**Figure 9:**
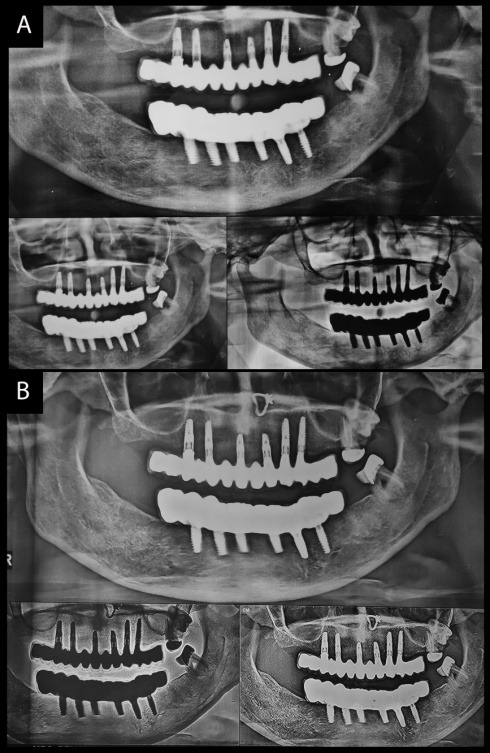
10-year follow-up for the completely edentulous patient.

**Figure 10:**
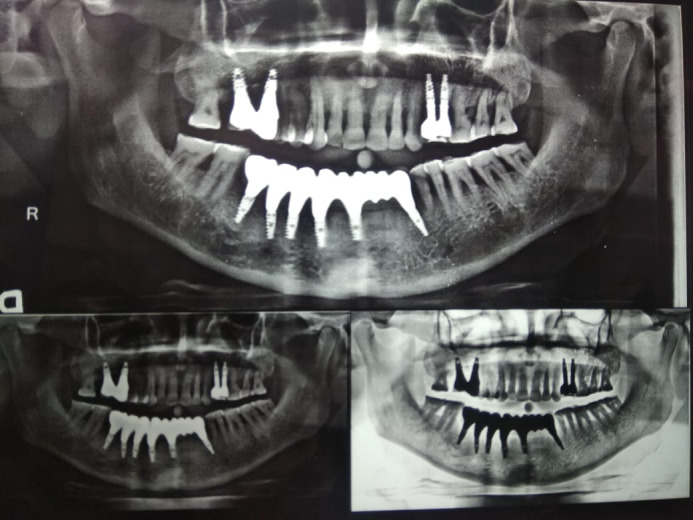
Follow-up for the partially edentulous patient.

Attachment loss around teeth in the partially edentulous patient increased further to around 3.5-4 mm. There was no implant loss, prosthesis complications like ceramic fracture and unscrewing of abutments in both patients (Figures 9 and 10).This could be attributed to follow-ups at regular intervals.

## Discussion

A clinically relevant periodontal prognosis model must be able to accurately predict the course of disease in a way that has a meaning to both the patient and the treating clinician, as well as to other dental professionals.^1^ Previous studies on periodontal prognosis have established that regardless of treatment, the most important modifier of periodontal disease progression is the patient.^2,3^ Due to patient-level modifiers such as genetics, smoking and diabetes, there will always be a subset of patients at increased risk of tooth loss, regardless of the treatment provided. To accurately predict a tooth prognosis, it might always be inaccurate. The patient also wants to know the future prospects of individual teeth and the ability and willingness to follow a comprehensive treatment plan. In our report, we rehabilitated two chronic sufferers of periodontitis by implants and saved teeth, which had a favourable prognosis. There is conflicting evidence on the outcomes of implants in periodontally compromised patients. Ormainer et al^4^ reported the efficacy of dental implant therapy in periodontally compromised patients who were followed for 9.5 years. They found that periodontal susceptibility resulted in increased bone loss but did not affect implant survival. Microbial aetiology is the cause of periodontitis. Several studies have shown that partially edentulous patients are at increased risk of cross-infection between periodontal and peri-implant sites.

In partially dentate periodontal maintenance patients with dental implants, a positive association between periodontal and peri-implant conditions was found after 10 years.^5^ Using multiple linear analyses, the authors determined that deeper mean full-mouth pocket depths and greater full-mouth attachment loss was associated around implants. In our report, both patients had chronic periodontitis and there were increases in both parameters. According to Klokkevold et al,^6^ a history of treated periodontitis does not appear to affect the implant survival rate as in our case, but it can negatively influence its success over a very long time. Renvert et al^7^ also reported that a history of periodontitis could be a contributing factor for peri-implantitis but stressed that the data to support this conclusion was very robust. According to a systematic review by Zangrando et al,^8^ implant prosthesis outcomes in periodontitis patients have satisfactory outcomes and high survival rates after 10 years of follow-up. However, several studies have stated that the severity of periodontitis appears to exert an effect on the rate of biological complications of dental implants. The data suggest lower implant survival, increased peri-implant bone loss and higher incidence of peri-implantitis.^9-11^ Studies have also reported that implants placed in aggressive periodontitis cases have lower survival rates and increased bone loss as compared to chronic periodontitis cases.^12,13^

Studies cannot be taken without caution as there are several heterogeneous factors, for example, implant system, surface characteristics, site of implant placement, protocols for bone augmentation, loading and follow-up periods from the baseline.^14^

The evidence for the optimal amount of keratinized mucosa is still controversial as studies have reported that it had no effect on bleeding on probing and pocket depth around implants. Future interventional studies are needed to confirm the impact of the keratinized width on the health of peri-implant tissues.^15^ In a recent systematic review, the evidence regarding this topic is limited.^16^

Supportive periodontal therapy is of paramount importance for the periodontal health of implants. It has been emphasized that residual pockets should be removed entirely after initial therapy because their presence might lead to biological complications. Recall visits by the patients provide periodontal control for clinicians. These visits form the basis of long-term success after implant placement and prevent recurrence of periodontitis. Both patients exhibited improvements in plaque control and their ability to perform better for long-term maintenance. Further follow-up with more patients with chronic periodontitis is needed with the control of a heterogeneous group to conclude that implants fail earlier than in the healthy group of patients. As in our case, both partially or completely edentulous patients rehabilitated with implants that were functioning well even after 10 years.

## Conflict of Interests

The authors declare no conflict(s) of interest related to the publication of this work.

## Authors’ Contributions

Clinical work, follow ups, design of article and intellect.

## Ethics Approval

None.
